# Weed target detection at seedling stage in paddy fields based on YOLOX

**DOI:** 10.1371/journal.pone.0294709

**Published:** 2023-12-13

**Authors:** Xiangwu Deng, Long Qi, Zhuwen Liu, Song Liang, Kunsong Gong, Guangjun Qiu

**Affiliations:** 1 College of Electronic Information Engineering, Guangdong University of Petrochemical Technology, Maoming, China; 2 College of Engineering, South China Agricultural University, Guangzhou, China; 3 Institute of Facility Agriculture of Guangdong Academy of Agricultural Sciences, Guangzhou, China; 4 Life Science and Technology School, Lingnan Normal University, Zhanjiang, China; Manipal Institute of Technology, INDIA

## Abstract

Weeds are one of the greatest threats to the growth of rice, and the loss of crops is greater in the early stage of rice growth. Traditional large-area spraying cannot selectively spray weeds and can easily cause herbicide waste and environmental pollution. To realize the transformation from large-area spraying to precision spraying in rice fields, it is necessary to quickly and efficiently detect the distribution of weeds. Benefiting from the rapid development of vision technology and deep learning, this study applies a computer vision method based on deep-learning-driven rice field weed target detection. To address the need to identify small dense targets at the rice seedling stage in paddy fields, this study propose a method for weed target detection based on YOLOX, which is composed of a CSPDarknet backbone network, a feature pyramid network (FPN) enhanced feature extraction network and a YOLO Head detector. The CSPDarknet backbone network extracts feature layers with dimensions of 80 pixels ⊆ 80 pixels, 40 pixels ⊆ 40 pixels and 20 pixels ⊆ 20 pixels. The FPN fuses the features from these three scales, and YOLO Head realizes the regression of the object classification and prediction boxes. In performance comparisons of different models, including YOLOv3, YOLOv4-tiny, YOLOv5-s, SSD and several models of the YOLOX series, namely, YOLOX-s, YOLOX-m, YOLOX-nano, and YOLOX-tiny, the results show that the YOLOX-tiny model performs best. The mAP, F1, and recall values from the YOLOX-tiny model are 0.980, 0.95, and 0.983, respectively. Meanwhile, the intermediate variable memory generated during the model calculation of YOLOX-tiny is only 259.62 MB, making it suitable for deployment in intelligent agricultural devices. However, although the YOLOX-tiny model is the best on the dataset in this paper, this is not true in general. The experimental results suggest that the method proposed in this paper can improve the model performance for the small target detection of sheltered weeds and dense weeds at the rice seedling stage in paddy fields. A weed target detection model suitable for embedded computing platforms is obtained by comparing different single-stage target detection models, thereby laying a foundation for the realization of unmanned targeted herbicide spraying performed by agricultural robots.

## Introduction

Weeds in rice fields compete with rice seedlings for light, water and soil nutrients, and provide a breeding environment for diseases and pests, which can seriously compromise the growth of rice, and lead to reductions in rice yield and quality. Weeding is also very labor-intensive work for farmers. Every year, Chinese farmers spend approximately 2–3 billion working days weeding. Even so, the annual grain loss caused by weeds in China still accounts for 13.4% of the total grain output, approximately 17, 500 thousand tons [1]. At present, there are many kinds of weed control methods, including manual weeding, chemical weeding, biological weeding, and mechanical weeding. Among them, chemical weeding is the most important method of weeding rice fields [2]. Chemical herbicides are commonly sprayed in rice fields on a large scale. However, due to the indiscriminate spraying of rice seedlings and weed-free areas, the effective utilization rate of herbicides is lower than 30% [3]. The excessive use of chemical herbicides in farmland will cause environmental pollution and herbicide damage to crops in the next season. Variable spraying guided by precision maps is a promising means of reducing pesticide waste. At present, farmland spraying technology mainly includes unmanned aerial vehicle (UAV) large-area spraying, spraying based on vehicle-mounted machine vision systems, and agricultural machinery for variable spraying guided by weed distribution density maps (Slaughter et al. 2008) [4]. Combining UAV platforms and deep learning algorithms to generate farmland weed distribution density maps shows promise as a new technical approach [5].

With the continuous development of modern agricultural production technology, machine vision technology and deep learning have been applied for weed target detection in precision agriculture [6, 7]. Traditional weed spraying systems spray pesticides regardless of the presence of weeds. In contrast, targeted spraying technology can distinguish between target objects for spraying and the paddy field background and adjust the level of spraying in accordance with the quantitative characteristics of the weed targets [8]. This can effectively reduce the use of pesticides, improve the quality of spraying operations and reduce environmental pollution. Weed target information detection technology is the basis of such targeted application technology. In the process of precise spraying, the weed target information acquisition unit identifies spraying targets and sends information to the controller to decide whether to spray herbicide.

Computer vision technology has the advantages of simple operation, low cost, a nondestructive nature, speed and accuracy, and the application of computer vision technology for rice field weed identification is an inevitable trend in the development of smart agriculture [9]. The traditional methods of visually detecting weed targets mainly rely on the manual extraction of the significant features of weeds, such as color [10], shape [11], texture [12], and hyperspectral features [13], to distinguish between weeds and crops. However, hyperspectral equipment is not suitable for general use due to its high price and complicated subsequent data processing.

In agricultural scenarios, because of the complex field environment and the different sizes of targets, certain difficulties are encountered in target detection. Especially for small targets, the detection effect is not ideal due to the weak features of the targets contained in the image and the lack of feature information [14]. In particular, the small row spacing and the presence of overlapping clusters of weeds in paddy fields increase the difficulty of weed identification.

An expert system has been built for crop and weed species identification based on color characteristics [15]. In addition, moment invariants and shape characteristics have been used to identify and classify weeds [16]. In another study, color segmentation and morphological operations were first performed on weed images, and 7 invariant moments and 6 geometric shape features of weeds were extracted for weed identification [17]. A different work focused on identifying oat and dandelion cotyledons under overlapping conditions, and the differences of oat and dandelion leaf texture characteristics were analyzed and compared [18].

In recent years, with the popularization of unmanned aerial vehicles (UAVs) for plant protection, weed area detection based on UAVs has become a popular topic of research. In one study, UAVs were used to acquire spectral images of sunflower and corn at the 4–6 leaf stage [19]. Ten features were extracted through a clustering selection and segmentation algorithm, and the weed coverage area was detected using supervised machine learning to determine the weed area position. In another study, corn images were collected with a spectral camera and an RGB camera at three heights of 30, 60, and 100 meters by an unmanned aerial vehicle [20], and corn crop rows and weeds in the crop rows were detected using an object-based area projection algorithm to obtain a weed area coverage map. Borra Serrano et al. collected visible light and near-infrared sunflower field images at 30, 60, and 100 meters using unmanned aerial vehicles [21] and resampled the collected images. These authors tested the detection of weed areas in both the directly collected images and the resampled images, showing that the detection effects were good for the directly collected images at 30 meters and the resampled images at 60 and 100 meters. When used to detect the weed area position, area projection methods may produce large errors, making it impossible to detect weed target areas accurately. However, the approach of detecting the weed area position by means of UAVs is mainly aimed at crops with large row spacings. The two methods above cannot solve the technical problems facing weed region positioning under more challenging conditions. Indeed, most methods perform poorly when applied to images with complex backgrounds such as images of paddy fields with small row spacing and overlapping clusters of weeds.

With the rapid development of artificial intelligence technology, deep learning and machine vision technology are gradually being applied to agricultural research, helping researchers solve many problems in the agricultural field and further promoting the transformation of traditional agriculture into intelligent agriculture [22].

Traditional target detection methods using machine vision technology require humans to design the features extracted in each step, making them subjective to some extent. Based on vision technology and deep learning, convolutional neural networks (CNNs) have been introduced for target detection [23], which has become the mainstream approach to target detection tasks at present [24]. Deep-learning-based target detection technology can effectively solve the problem of weed target recognition. It has a high recognition rate, returns accurate location information, can achieve automatic feature extraction with no need for manual design, and can extract deep abstract features that are difficult to specify manually. It can be applied in a wide range of recognition scenarios, offers strong universality and robustness, and supports end-to-end integrated training [25].

There are two main approaches to small target detection: one is a single-stage target detection network model, as represented by You Only Look Once (YOLO) [26], and the other is a two-stage target detection network model, as represented by Region CNN (RCNN) [27], Fast RCNN [28], and Faster RCNN [29].

Compared with two-stage target detection network models, a single-stage target detection algorithm omits the generation of candidate regions, and focuses only on the tasks of feature extraction, target classification and regression.

A single-stage target detection algorithm identifies the detection results for an image through one step of processing on the input image. The relatively well-established single-stage algorithms include the YOLO series [26] and the single shot multibox detector (SSD) series [30]. The SSD algorithm proposed by Liu’s team in 2016 has the speed of YOLO and the accuracy of RCNN.

In recent years, in applied engineering research on target detection, the YOLO series has been favored by researchers because of its fast response, high precision, simple structure and easy deployment. The YOLO algorithm was proposed by the Redmon team in 2016. It condenses the two stages of the RCNN series into one stage. It does not require a separate step for classification but instead directly obtains the target category and location information from the input image. Compared with traditional detection methods, the YOLO process is simpler, faster, and more suitable for real-time applications. It uses a CNN to directly generate prediction boxes and categories. In 2017, the Redmon team proposed YOLOv2, an updated version of YOLOv1. Compared with YOLOv1, it uses the k-means clustering algorithm to generate an a priori frame, and also uses a new backbone network, which significantly improves the target detection accuracy compared with YOLOv1. Then, in 2018, the Redmon team presented YOLOv3 [31], which further adds a feature pyramid network (FPN) [32] based on YOLOv2. Its main aim is to splice features of different depths and output feature maps of different scales for classification prediction, use the gradient descent algorithm to find the optimal solution, and then use non-maximum suppression (NMS) to remove redundant boxes to finally produce the output result map. YOLOv4 [33] came out in 2020, and soon, YOLOv5 [34] will also be released. These two most recent versions further optimize the performance and structure of YOLO from different directions.

Traditional target detection algorithms offer very accurate, rapid and reliable detection of large-scale targets, but for the detection of small targets, the desired results have not yet been achieved. For the small targets detection of weeds in rice fields at the seedling stage, the training speed and prediction speed of a single-stage target detection algorithm will be superior, but some problems will also be encountered: because small targets cover only a few pixels in their divided boundary boxes, the texture and resolution of such objects are very low, which means that there are very few features that can be extracted. Moreover, many target detection algorithms use only high-level features for prediction. The semantic information carried by high-level features is rich, but their resolution is low, so the target location derived from such features is rough. In a deep network, a small object may correspond to a single output image pixel in the final high-level feature map, thus, its features are lost. In contrast, low-level features carry relatively little semantic information, but they can provide the accurate target location, which is helpful for small target detection [31, 32]. However, the semantic information of low-level features in a CNN is not effectively used. With increasing convolutional layer depth, the feature information of small targets will become fuzzy or will be lost. At the same time, the receptive field of the feature map will be large, which is not conducive to the detection of small targets, leading to problems of missing and incorrect detection.

YOLOX [35] was proposed in 2021 by reorganizing and optimizing the excellent structures in previous versions of YOLO to achieve better performance. YOLOv3, YOLOv4 and YOLOv5 all have the problem that the model generalization ability is poor due to the need to manually designate positive and negative samples. Compared with other YOLO series algorithms, YOLOX has the best detection speed and accuracy in target detection. Alternanthera philoxeroides is a perennial herb of the genus Amaranthaceae, that originated in South America. After invading farmland, it competes with crops for water, light, fertilizer, and growth space. As a result, the yields of a variety of grains, oil crops, and cash crops are reduced to varying degrees, especially rice [36]. Due to the small row spacing of rice seedlings in paddy fields, weeds and seedlings can easily cover each other when growing in a limited space. Moreover, weeds in paddy fields have different germination periods. Hence, Alternanthera philoxeroides weeds of different sizes can appear in a field at the same time due to their different growth periods. Alternanthera philoxeroides is a broad-leaf weed. This is a relevant qualification because it often guides herbicide selection. In this study, taking Alternanthera philoxeroides as an example, a small target detection algorithm for weeds in rice fields based on YOLOX is proposed to solve the problem of detecting the locations of small and partially occluded weed targets against the complex background of a rice field.

With the expansion of deep learning methods for object detection, related technologies are gradually providing embedded devices with stronger computing and analysis capabilities. In practical applications of the selective spraying of chemical herbicides, it is necessary to deploy small weed target detection models on embedded computing platforms [37]. By making use of "edge computing" technology for advanced AI, a deep learning algorithm can be combined with a visual camera to provide location information for targeted herbicide spraying operation. Therefore, the exploration of a small weed target detection model with high detection accuracy, a fast operation speed, and easy generalization has broad research value and application prospects. In accordance with the characteristics of rice-field-associated weeds and paddy field environments, machine vision and deep learning are used in this study to carry out research on relevant methods of weed target location detection in rice fields. This study solves the technical problem of target location detection for rice associated weeds against the complex background of a rice field, and provides technical support for targeted herbicide spraying in rice fields.

## Material and methods

### Image acquisition

**Fig 1:**
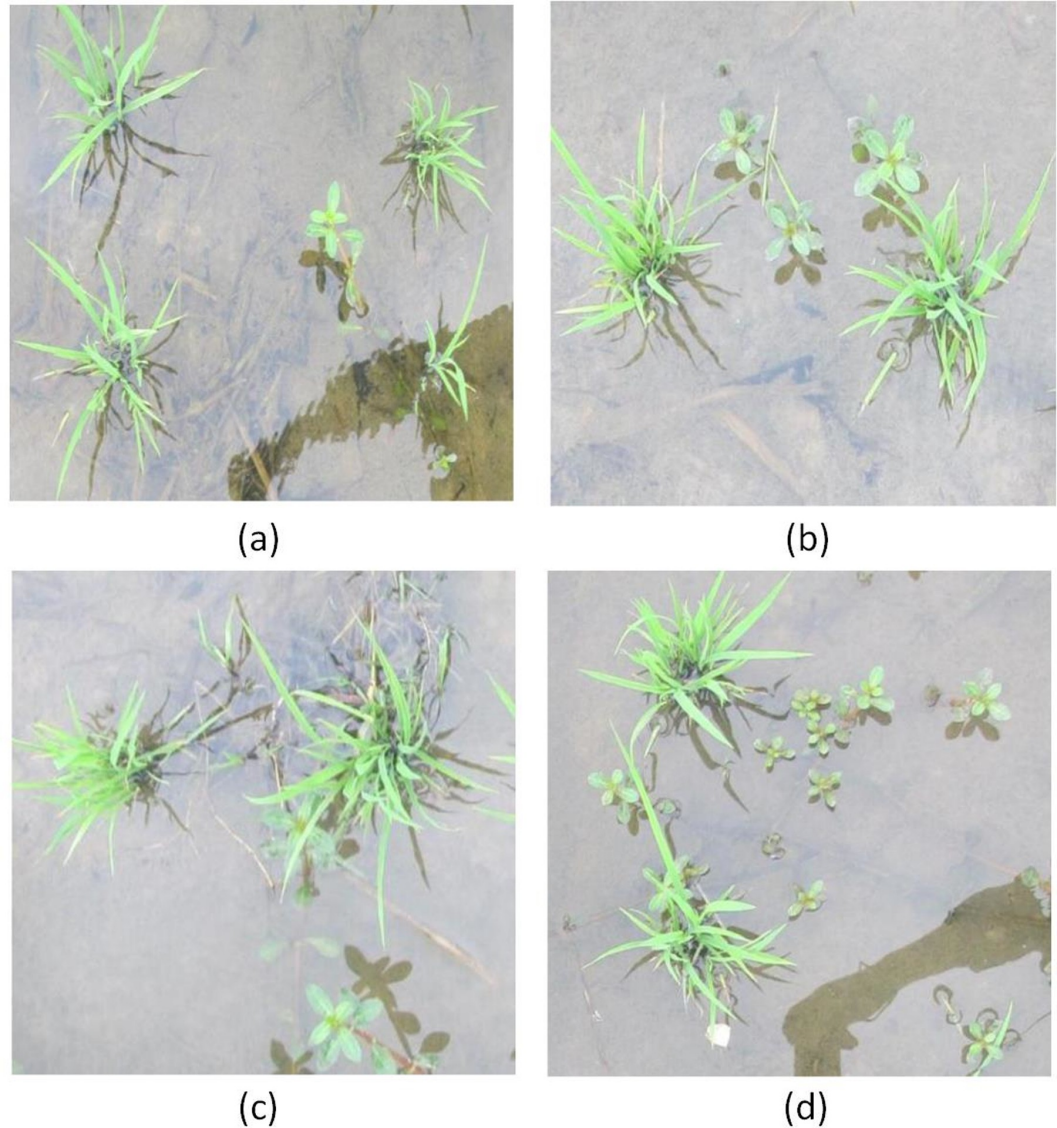
Examples of weed target detection based on manually labeled regions: (a) not occluded; (b) multiple weed targets; (c) slight occlusion; (d) dense weeds.

The rice field weed target detection samples were images collected 15 days after the transplanting in 2018 and 2019 when the seedlings were not sealed. Image acquisition was carried out by hand with a Canon SLR digital camera, and the specific equipment was an IXUS 1000 HS (EF-S 36–360 mm f/3.4–5.6 IS STM). The focusing mode was set to: automatic intelligent focusing, and the image resolution was 640 × 480 pixels. Image acquisition was carried out under outdoor natural light conditions. The collection position was 60–80 cm away from the weeds, and the camera lens was perpendicular to the water surface of the paddy field. In total, 358 images were obtained using the image acquisition platform in the field. The images were resized to 500 pixels × 500 pixels. Each image contained multiple weed targets. Examples of weed target detection from the seedlings and weeds in the collected images are shown in Fig 1. To avoid the impact of excessively strong light on weed image acquisition, cloudy weather was mainly selected for shooting. The labeled weed image samples were randomly divided into training samples, test samples, and validation samples, where the last were part of the training samples. Specifically, 80% were training samples, 20% of which were validation samples, and the remaining 20% were test samples. The training samples were used to train the model and determine its parameters, the validation samples were used to determine the network structure and adjust the parameters of the model, and the test samples were used to test the generalization ability of the model.

### Methods

**Fig 2:**
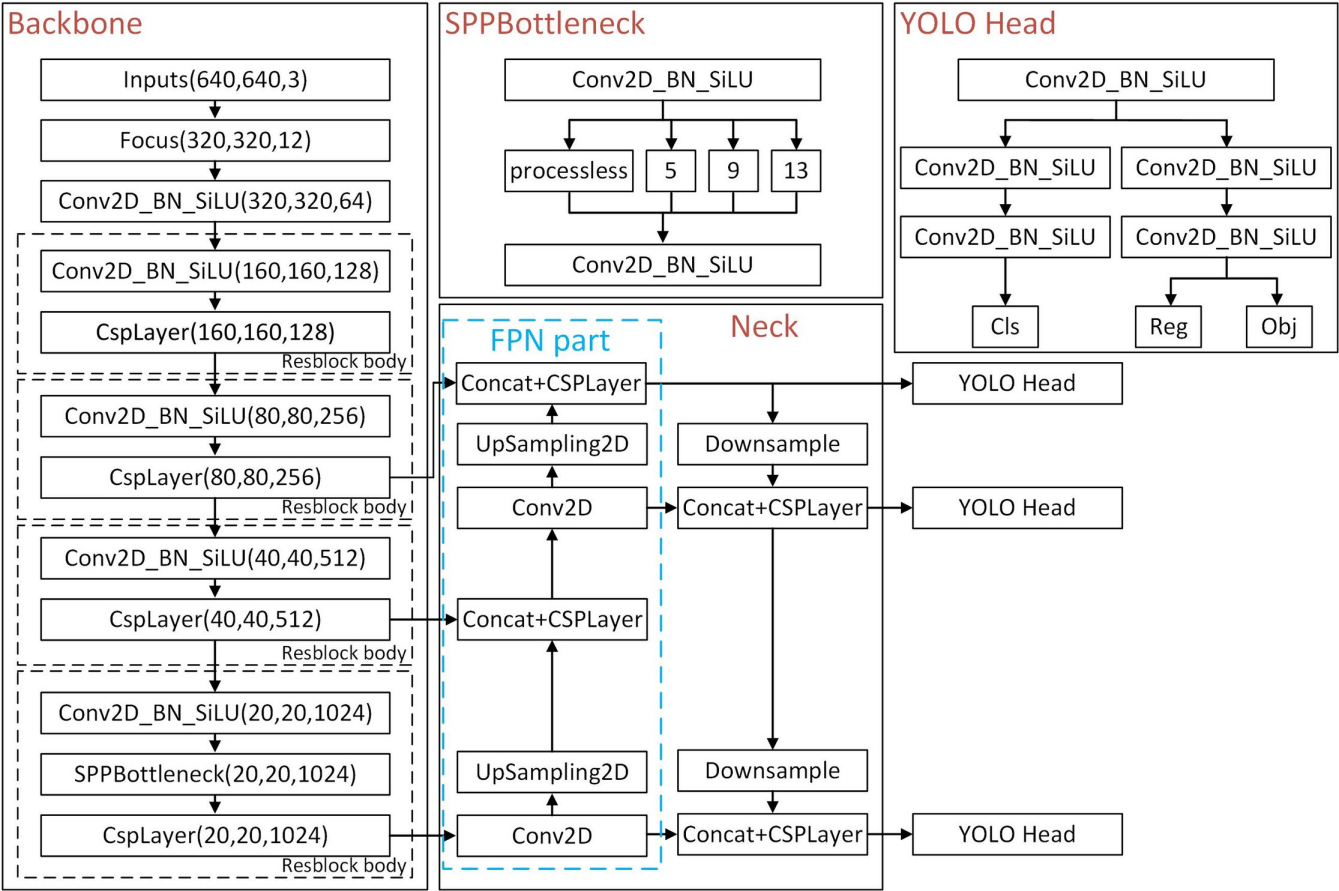
YOLOX model structure diagram.

We review the YOLOX structure from [35] in this section. The YOLOX network is composed of a backbone, a neck and a YOLO Head, and the model variants in the YOLOX series include the S, M, L and X models and the lightweight Tiny and Nano models. The YOLOX network structure is shown in Fig 2. YOLOX uses CSPDarknet as the backbone network to extract features and introduces an FPN to output features at different scales to improve the accuracy of small target detection. First, features are extracted from the input image by CSPDarknet. The extracted features represent the feature set of the input image. In the backbone, three feature layers are obtained for the next step of network construction, which are called effective feature layers. The three effective feature layers obtained in CSPDarknet are then fused. The purpose of feature fusion is to combine multiscale feature information. In the FPN part, the obtained effective feature layers are used to continue to extract features. Through feature extraction, upsampling and downsampling, and feature fusion in the CSPDarknet and FPN networks, three enhanced effective feature layers are obtained for target detection through the YOLO Head detector. The YOLO Head is divided into two parts to separately realize the classification of objects and the regression of prediction boxes, which are integrated in the final prediction.

The backbone in YOLOX is based on the CSPDarknet network and accepts input images with of three channels of 640 pixels ⊆ 640 pixels. YOLOX outputs feature maps at three scales, namely, 80 pixels ⊆ 80 pixels, 40 pixels ⊆ 40 pixels, and 20 pixels ⊆ 20 pixels, after feature extraction through the backbone network, and the feature information at different scales is passed to the neck network for feature fusion after convolution and stacking, as shown in Eq (1). Here, Pi refers to the feature map transferred to the neck after CSPDarknet sampling, and *C*_*i*_ refers to the hierarchical features in the CSPDarknet network.


$$P_{i}、\;\;\;P_{i+1}、\;\ldots \;\;\;\ldots 、\;\;\;P_{i+n}\;=\;F\left(C_{i}、\;\;\;C_{i+1}、\ldots \;\;\;\ldots 、\;\;\;C_{i+n}\right)$$ (1)

The extracted feature maps at the three scales are input into the prediction network to analyze the feature semantics. This network predicts the classification, location, and confidence of the feature information, and selects a prior box with the highest confidence as the final prediction result after NMS. The loss function used in the prediction network of YOLOX is a weighted sum of the classification loss function and the boundary regression loss function. The Eqs are shown as follows (2–4).


$$C_{ij}\;=\;L_{ij}^{cls}\;+\;\lambda L_{ij}^{reg}$$ (2)

$$L_{reg}\;=\;-\;log\left(IoU\left(B_{gt},\;B_{pred\;}\right)\right)$$ (3)

$$L_{cls}\;=\;-\sum_{i=1}^{n}\left(t_{i}\left(log\left(p_{i}\right)\;+\;\left(1-t_{i}\right)log\left(1\;-\;p_{i}\right)\right)\right.$$ (4)

Here, λ is a balance coefficient and $L_{ij}^{cls}$ is the classification loss function. First, the sigmoid function is used to predict the classification confidence; then, binary cross entropy is used to calculate the classification loss function. $L_{ij}^{reg}$ is the boundary regression loss function, which is used to select the best prediction box based on the IoU, and *C*_*ij*_ is the total loss cost of the whole model. *B*_*gt*_ is the true bounding box, and *B*_*pred*_ is the predicted bounding box. The IoU is calculated as the ratio of the intersection and union of the predicted and true bounding boxes. In this paper, the IoU threshold is set to 0.5. When the IoU value is greater than this threshold, this instance is determined to be a true positive (*TP*), and otherwise, it is determined to be a false positive (*FP*).

#### Data enhancement and rice field weed detection

YOLOX adopts the mosaic data enhancement mode at the input end [38]. The mosaic data enhancement method treat 4 images as a group for splicing. The main advantage of the mosaic method is that it can enrich the number of objects to be detected. By randomly scaling and then randomly distributing the input data, the mosaic method greatly enriches the detection dataset. In particular, random scaling adds many small targets, making the network more robust. At the same time, when batch normalization is used for calculation [39], the data from four images are calculated at one time, so the minibatch size does not need to be very large.

**Fig 3:**
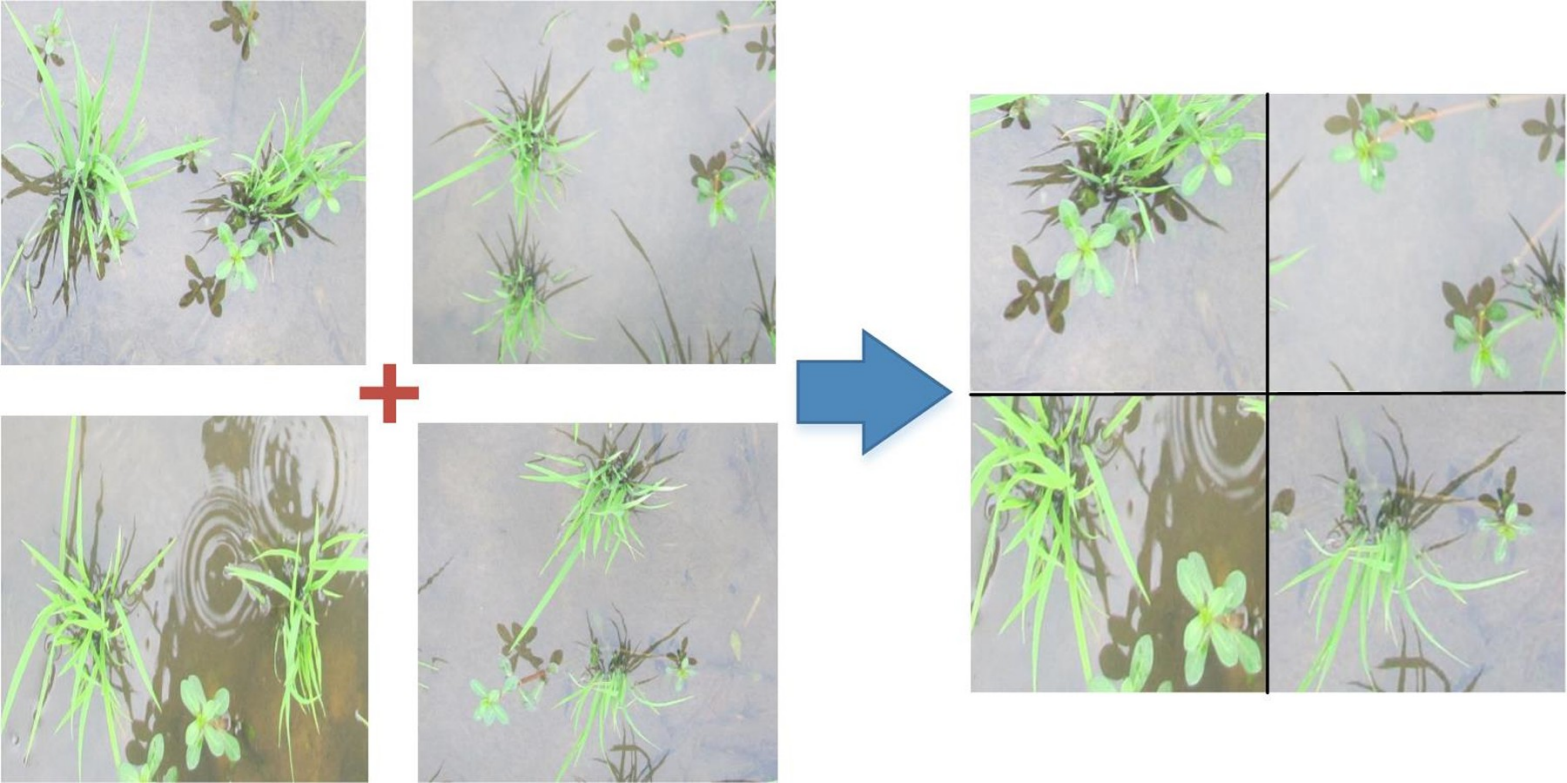
Mosaic data enhancement diagram.

A group of four paddy field weed images at the seedling stage was randomly selected from the dataset; then, the methods of random scaling, cutting, and arranging were used to splice the four images. The whole mosaic data enhancement process is shown in Fig 3. Compared with any single original image of Alternanthera philoxeroides, the synthesized image contains more weed targets, which can help to overcome the learning difficulties caused by an imbalance of positive and negative samples of small targets.

#### Neck network

In the backbone feature extraction network of the YOLOX model, CSPDarknet, the simple stack of original residual blocks is split into two parts, namely, Shortconv and Mainconv. The Shortconv part has a large residual edge, which bypasses many residual structures. It is directly connected to the last layer after the 1 × 1 convolution operation of Conv2D_BN_SiLN (CBS). After the CBS (1 × 1) convolution operation in CSPDarknet, multiple residual cells are stacked in sequence. The values of n are 1, 2, 8, 8, and 4, and each residual structure is composed of several residual units. A CBS (1 × 1) convolution is used to adjust the number of channels, and then a CBS (3 × 3) convolution is performed to enhance feature extraction; then, the output and a small residual edge are added, and one more CBS (1 × 1) convolution is performed. At this time, the number of channels is adjusted to be consistent with Shortconv. Finally, Shortconv and Mainconv are stacked. Another CBS (1 × 1) convolution is performed for the final channel integration before the features are output.

**Fig 4:**
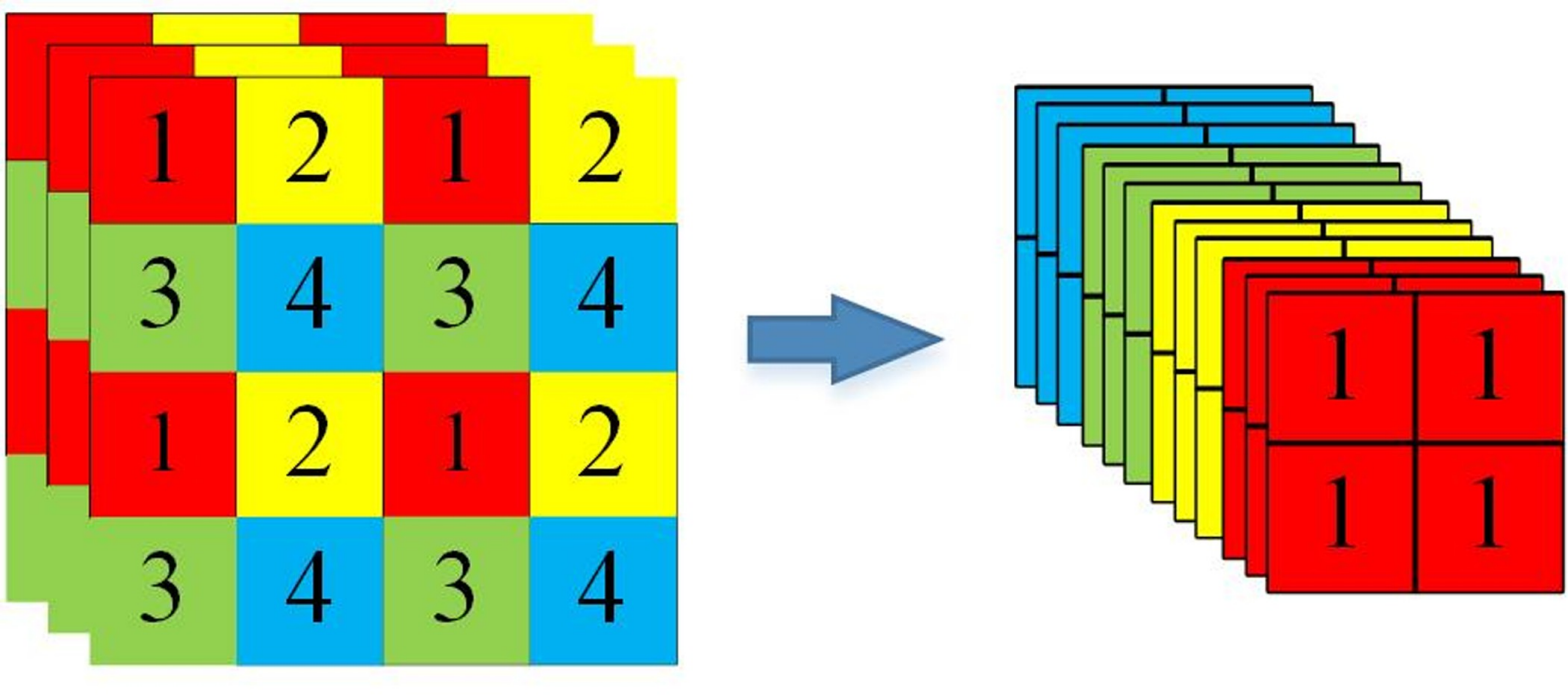
Focus network structure.

The backbone network uses a focus network structure to divide the feature points in the image. The specific operation takes a value for every other pixel in the image. The principle is shown in Fig 4. The feature layers of the corresponding regions of each color are stacked. At this time, the width and height information of the compressed image is concentrated into the channel information. Subsequently, the input channels are expanded by a factor of four. Thus, an image with dimensions of 608 × 608 × 3 is expanded to 304× 304 × 12.

**Fig 5:**
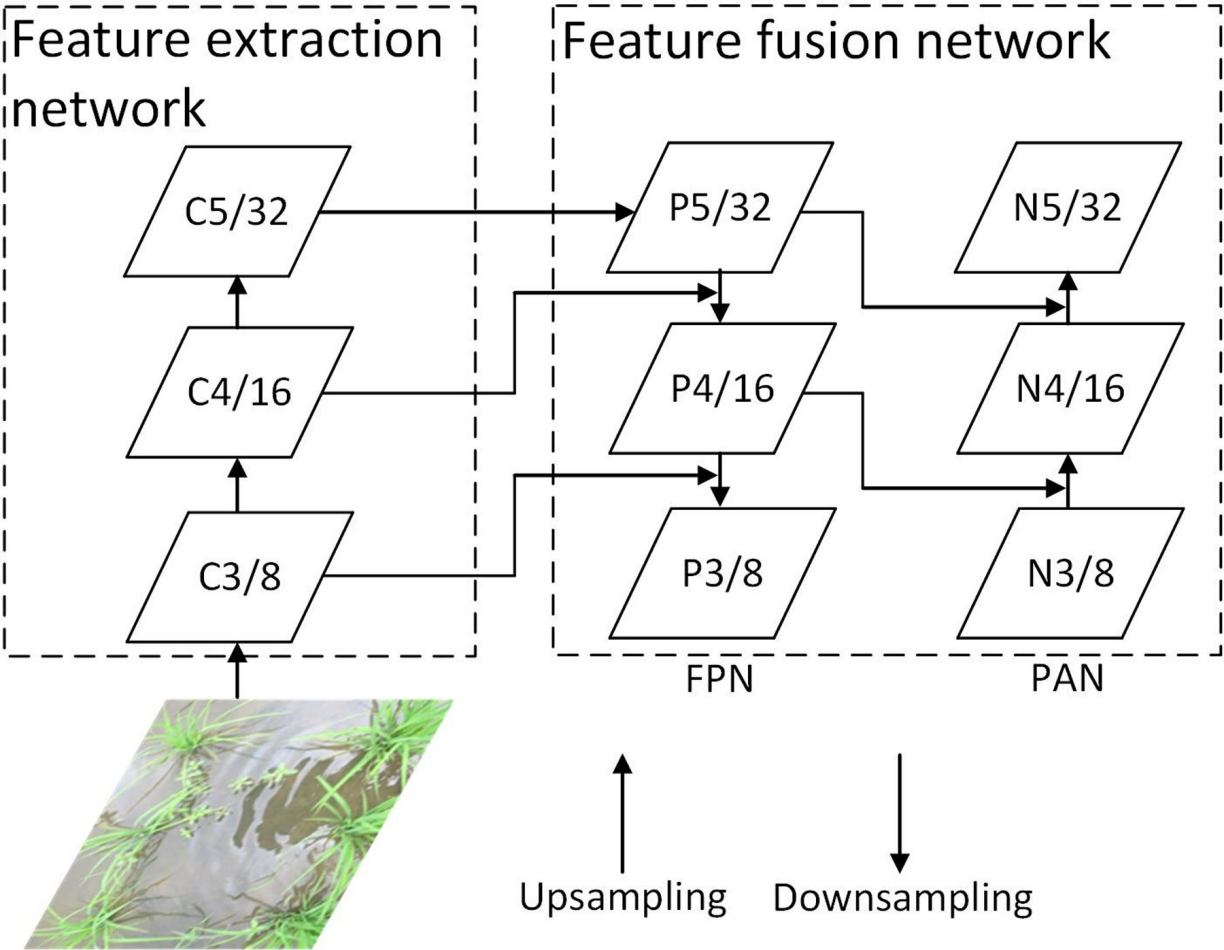
FPN+PAN structure in YOLOX.

In the neck network structure, YOLOX and the YOLOv3 baseline both adopt an FPN structure for integration. As shown in Fig 5, the FPN transfers and fuses the feature information of the upper layers from top to bottom by means of upsampling to obtain the feature map for prediction. In YOLOv4, YOLOv5, YOLOX-S, YOLOX-L and other versions, an FPN+PAN structure is adopted [40, 41]. A bottom-up feature pyramid (PAN) is added behind the FPN layer. In this way, the FPN layer can capture strong semantic features from top to bottom, whereas the feature pyramid can capture strong positioning features from bottom to top, and the parameters of the different detection layers are aggregated from different backbone layers.

### Evaluation metrics

To better evaluate the detection performance of the target detection model for weeds in rice fields at the seedling stage, the recognition precision, recall, harmonic mean (F1), average precision, and mean average precision (mAP) are used as evaluation indicators in this study. The specific definitions are presented subsequently.

The precision (*P*) is defined as the ratio of the number of positive classes predicted to be positive to the number of all predicted positive classes. Its mathematical expression is shown in Eq (5).


$$P\;=\;\frac{TP}{TP\;+\;FP}\;\times \;100\text{\%}$$ (5)

The recall (*R*) is defined as the ratio of the number of positive classes predicted to be positive to the number of all real positive classes. Its mathematical expression is shown in Eq (6).


$$R\;=\;\frac{TP}{TP\;+\;FN}\;\times \;100\text{\%}$$ (6)

Here, *TP* indicates that the predicted result is Alternanthera philoxeroides and this prediction is correct; *FP* indicates that the predicted result is Alternanthera philoxeroides, but the object actually is not Alternanthera philoxeroides, i.e., the result is a false detection; a false negative (*FN*) means that the predicted result is not Alternanthera philoxeroides, but the object actually is Alternanthera philoxeroides, i.e., it is not detected. The harmonic mean (F1) strikes a balance between the precision P and the recall R. The closer F1 is to 1, the better the model is optimized. Its mathematical expression is shown in Eq (7). If there are no weeds in the image, TP = 0, and FP = 0 or FP > 0. The model makes no predictions when P = 1, R = 0, and F1 = 0.


$$F1\;=\;\frac{2\;\times \;P\;\times \;R}{P\;+\;R}$$ (7)

The average precision (*AP*) represents the comprehensive effect of the precision and recall, which is reflected by the size of the area under the P-R curve. It is used to evaluate the performance of the model for the single detection category of Alternanthera philoxeroides, and reflects the overall performance of the model. Its mathematical expression is shown in Eq (8).


$$AP\;=\;\int_{0}^{1}P(R)dR$$ (8)

The mean average precision (*mAP*) is the average of the average precision values for all target categories. Its mathematical expression is shown in Eq (9).


$$mAP\;=\;\frac{\sum_{i=1}^{n}AP_{i}}{N}$$ (9)

In this equation, *N* represents the total number of target categories. In this test, there is only one category; thus, *N* = 1, and the *AP* value is consistent with the *mAP* value.

The detection speed refers to the number (frames) of weed images in the rice field seedling stage recognized per second, in frames per second (FPS). The detection speed is inversely proportional to the recognition time. The higher its value is, the shorter the recognition time for a single image is, and the faster the algorithm runs.

## Results and discussion

### Training parameters

A simple cross validation method is used in this article. First, we randomly divide the sample data into two parts (80% as the training set and 20% as the test set), then use the training set to train the model and validate the model and parameters on the test set. In deep learning, each epoch updates the weight and bias of the model to better adapt to the training data. Through multiple epochs of training, the model can gradually grasp the patterns and patterns in the data, thereby improving the accuracy of prediction. Training a model is an iterative process, and through multiple epochs of training, the model can gradually improve accuracy and performance. Each epoch updates the weight and bias of the model to better adapt to the training data. Through multiple epochs of training, the model can gradually grasp the patterns and patterns in the data, thereby improving the accuracy of prediction. Next, we shuffle the samples and reselect the training and test sets to continue training and testing the model. Finally, we choose a loss function to evaluate the optimal model and parameters.

The model training hardware environment used in this study was as follows: an I5-7 generation 4-core processor, 12 GB of memory, and a GTX 1080 Ti graphics card (11 GB) to accelerate image processing. All work related to AI model training in this paper was carried out on a computer, not involving any the ’edge computing’. The NVIDIA GeForce GTX 1080 Ti graphics card uses 250 W of power, which is higher than the power consumption of an embedded hardware platform; for example, that of the Jetson TX2 is 7.5–15 W. The software environment was as follows: the operating system was Windows 10, and the YOLOX model was trained using PyTorch. Pretrained YOLOX weights based on the VOC dataset were used to train the model through transfer learning.

The parameter optimizer used in the model training process was a random gradient descent algorithm. The learning rate was set to 0.002. The default minimum learning rate of the model was 0.01 of the maximum learning rate, and the momentum was 0.937. The whole training process was divided into two stages: freezing the trunk parameters for training, and then unfrozen training. During the frozen training stage, the batch mode learning method was applied. The batch size was set to 32, and the initial epoch was 0. In total 50 epochs were performed. After thawing, the training batch size was set to 16, and the whole training process was completed after 250 epochs.

**Fig 6:**
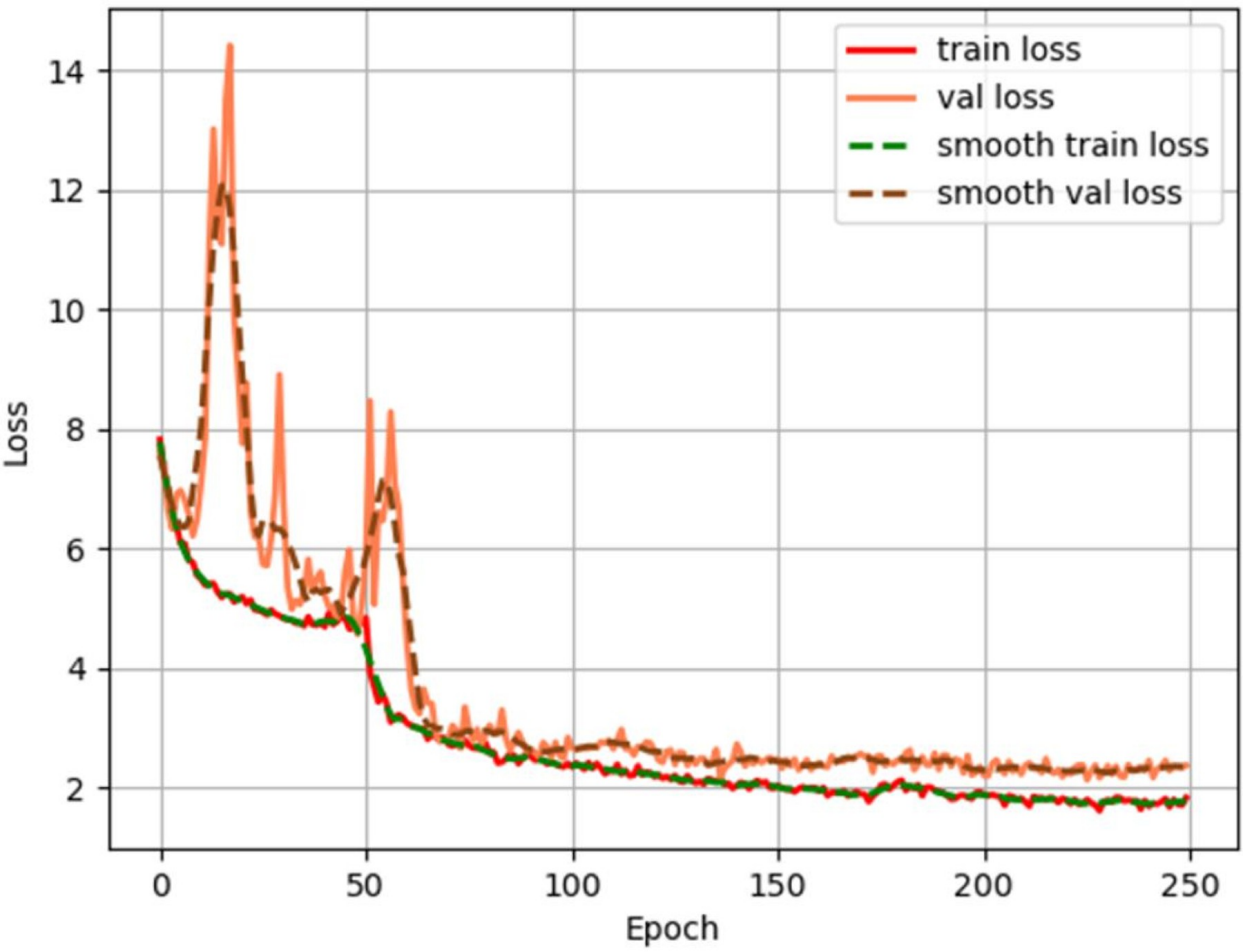
YOLOX-tiny loss curve.

In Fig 6, “train loss” denotes the curve showing the loss in each epoch on the training set, and “val loss” denotes the loss curve on the verification set. Similarly, “smooth train loss” and “smooth val loss” denote the results of applying smoothing to the loss curves. When the loss curve converged to a flat line and no longer declines, iteration was stopped, and the effect of the model was tested. By observing the loss curve, we could preliminarily solve the problems of underfitting and overfitting of the model by selecting an appropriate learning rate, batch size and number of iterations.

As shown in the figure, when training started, the loss on the verification set was very large, and it slowly converged while exhibiting oscillation. After the 50th epoch, the backbone network of the algorithm was unfrozen; subsequently, the loss on the verification set initially became larger again, and then slowly converged to a flat state.

### Comparison of the results

Although the two-stage target detection strategy offers greatly improved accuracy and speed compared with traditional target recognition algorithms, the detection efficiency is not high. This kind of algorithm needs to obtain a large number of candidate regions in advance, resulting in a high computational cost [39] and an inability to meet the needs of detection speed in practical applications. In contrast, the single-stage target detection models of the YOLO series are fast and small, making them more suitable for embedded computing platforms. Therefore, this paper mainly compares the performance of other single-stage target detection models (YOLOv3, YOLOv4 tiny, YOLOv5-s and SSD) with that of the YOLOX series. This study mainly explores small models for weed target detection applicable on embedded hardware platforms. The YOLOX-L and YOLOX-X models have more parameters than YOLO-m and YOLOX-s [25] and far more than the lightweight models YOLOX-nano and YOLOX-tiny; therefore, only YOLO-m, YOLOX-s, YOLOX-nano, and YOLOX-tiny were selected. In this experiment, 8 target detection models were trained, namely, YOLOv3, YOLOv4 tiny, and YOLOv5-s of the YOLO series; YOLOX-m, YOLOX-s, YOLOX-tiny, and YOLO-nano models as improved variants of the YOLO algorithm; and the SSD network, a classical algorithm model. Then, the performance and scale of each model were compared and analyzed.

**Fig 7:**
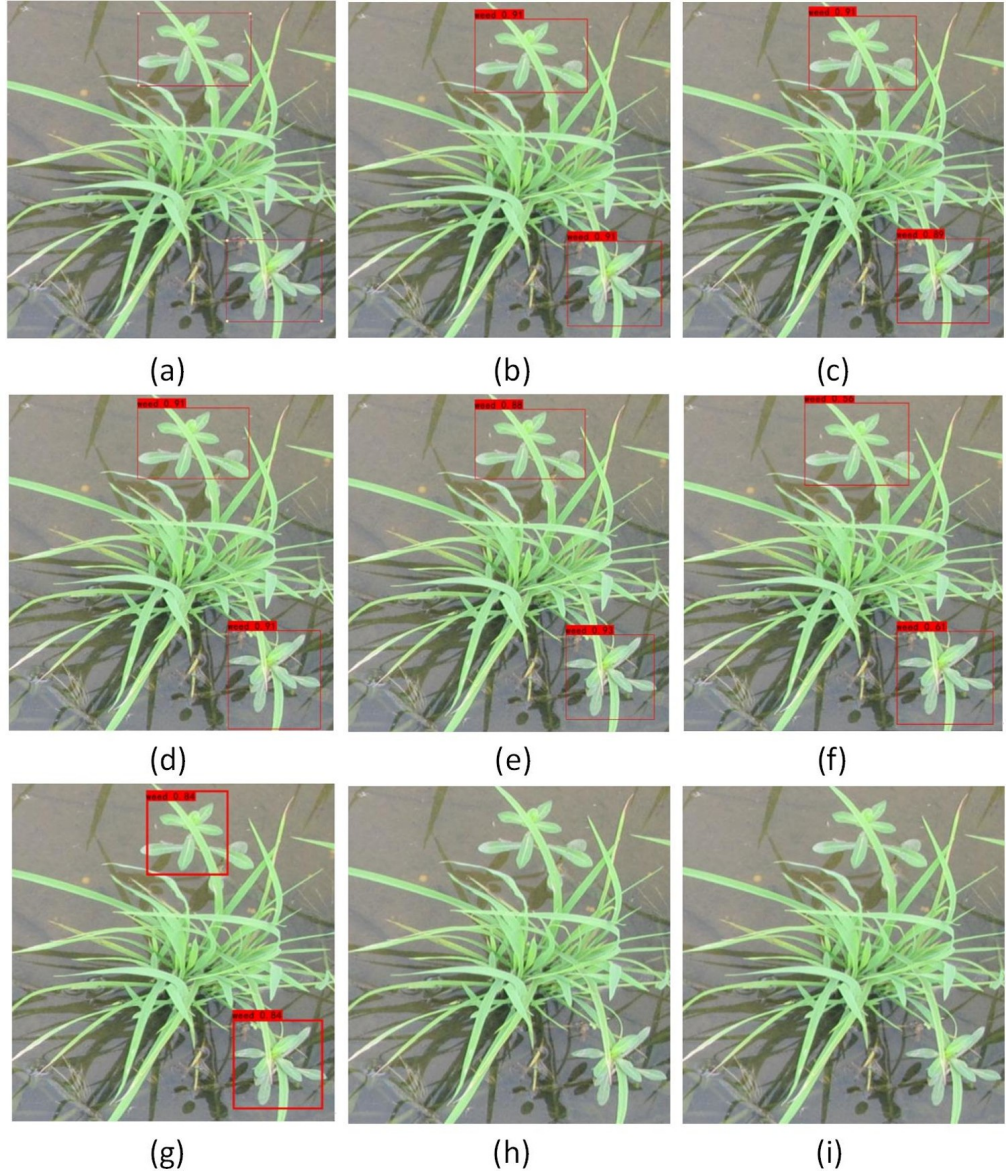
Comparison of the detection results of different models under slight shading. (a) target label; (b) YOLOX-tiny; (c) YOLOX-nano; (d) YOLOX-s; (e) YOLOX-m; (f) YOLOv5-s; (g) SSD; (h) YOLOv4-tiny; (i) YOLOv3.

As shown in Fig 7, only the YOLOv3 and YOLOv4-tiny algorithm models could not detect weeds when seedlings and weeds were slightly blocked; other models could detect the weed locations. However, the predicted frames of the YOLOv5-s and SSD algorithms exhibited certain errors compared with the real frames, and the confidence of the predicted frames was low. In comparision, the YOLOX series algorithms yielded good detection results.

**Fig 8:**
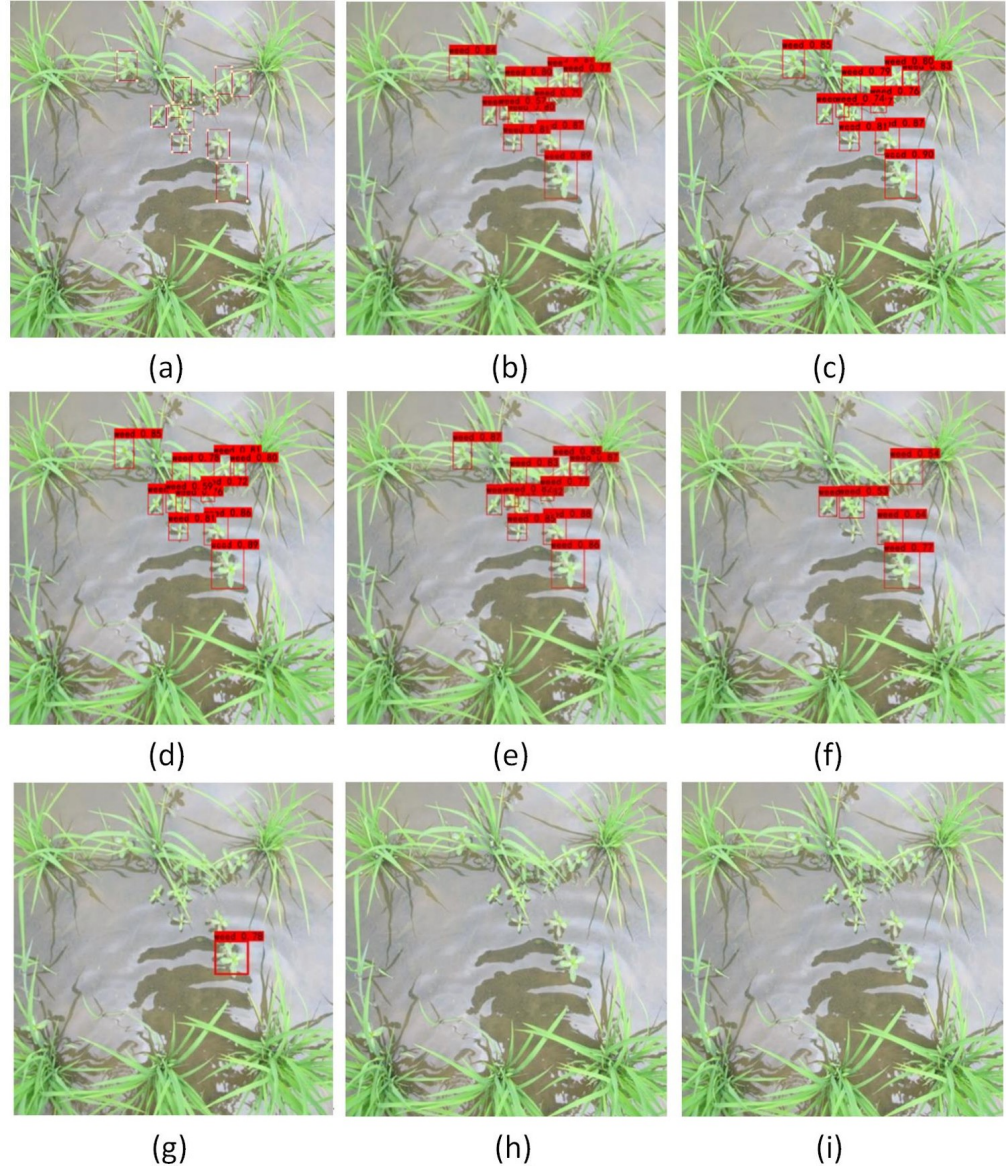
The test results of different models under dense weeds. (a) target label; (b) YOLOX-tiny; (c) YOLOX-nano; (d) YOLOX-s; (e) YOLOX-m; (f) YOLOv5-s; (g) SSD; (h) YOLOv4-tiny; (i) YOLOv3.

Fig 8 shows that the detection effect of the YOLOX models is very good when seedlings and weeds are sheltered from each other and weeds are closely associated with each other at different scales. There is not much difference in the detection effect between the YOLOX-m and YOLOX-s models, which have a larger network scale, and the lightweight YOLOX-tiny and YOLOX-nano models. The next best-performing model is the YOLOv5-s model. The YOLOv5 model has a certain detection ability for dense weeds of different shapes and sizes, but it does not perform well in dealing with the mutual shielding of seedlings and weeds. The classical SSD model cannot detect small weed targets when the weeds have different scales and high density, and also cannot detect the positions of weeds when seedlings and weeds occlude each other. The worst detection results were obtained with the YOLOv3 and YOLOv4 tiny models. The reason is that the weed dataset was too small to enable the YOLOv3 network to conduct sufficient weed feature extraction. Although the mosaic data enhancement technique was used in YOLOv4-tiny, it could still not achieve good results.

From the above performance comparisons of the different models, including YOLOv3, YOLOv4-tiny, YOLOv5-s, SSD and the YOLOX series (namely, YOLOX-s, YOLOX-m, YOLOX-nano and YOLOX-tiny), the results show that the YOLOX-tiny model of the YOLOX series performed best. The mAP, F1, and recall values for the YOLOX-tiny model were 0.980, 0.95, and 0.983, respectively. Meanwhile, the intermediate variable memory generated during the model calculation of YOLOX-tiny was also small: only 259.62 MB. Table 1 shows the mAP values in ascending order. It can be seen from this table that the s, m, nano and tiny model versions derived from YOLOX all perform better than the other models in weed position detection for Alternanthera philoxeroides, and YOLOX-tiny performs best in terms of mAP, F1, precision, and recall.

**Table 1:** Performance indicators of different network models.

Model	Backbone network	mAP	F1	Precision	Recall	FPS	Memory(MB)
YOLOv3	Darnet-53	0.661	0.39	**0.979**	0.246	15.3	440.40
YOLOv4-tiny	CSPDarkNet	0.791	0.70	0.909	0.571	**76.7**	**172.21**
YOLOv5-s	Focus+CSPDarknet	0.940	0.88	0.908	0.851	29.9	284.95
SSD	VGG	0.942	0.88	0.884	0.874	22.7	206.28
YOLOX-s	Focus+CSPDarknet	0.966	0.93	0.894	0.960	26.7	346.09
YOLOX-m	Focus+CSPDarknet	0.967	0.93	0.899	0.971	13.4	1497.75
YOLOX-nano	Focus+CSPDarknet	0.967	0.93	0.899	0.971	34.7	229.39
YOLOX-tiny	Focus+CSPDarknet	**0.980**	**0.95**	0.914	**0.983**	34.6	259.62

### Comparative analysis of model performance indicators

**Fig 9:**
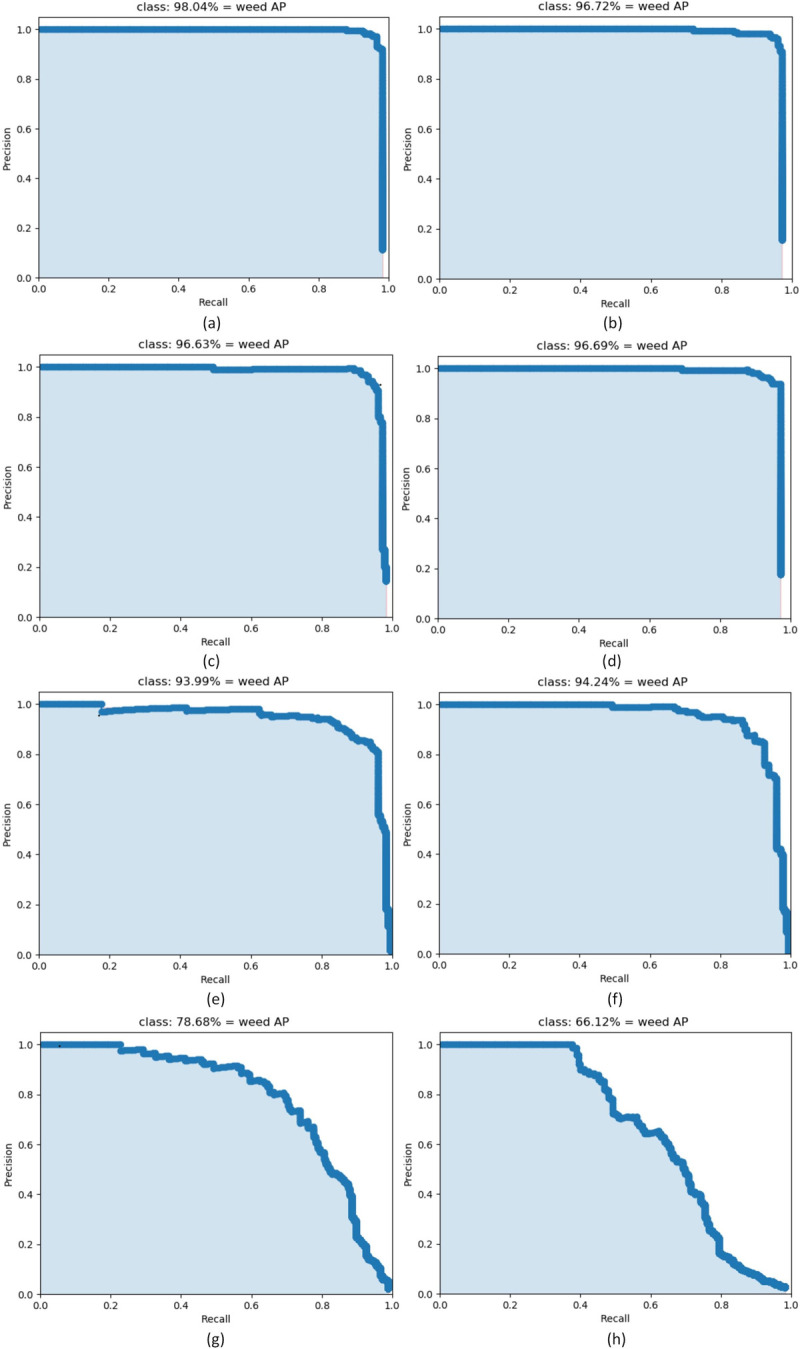
Comparison chart of PR curves of different models. (a) YOLOX-tiny; (b) YOLOX-nano; (c) YOLOX-s; (d) YOLOX-m; (e) YOLOv5-s; (f) SSD; (g) YOLOv4-tiny; (h) YOLOv3.

As shown in Fig 9, in the comparison of the PR curves of the different models, the abscissa represents the recall, and the ordinate represents the precision. This figure is called the PR figure for short. The curve shows the precision P when the recall R is fixed. The area at the bottom left of the PR graph represents the effect of the model on the dataset. It can be seen from this figure that the PR curves of the YOLOX algorithm models of all scales contains most of the plot area; thus, these models have a very good effect on this dataset. The YOLOv5 and SSD networks also have good effects, whereas the YOLOv4-tiny and YOLOv3 models have poor effects.

**Fig 10:**
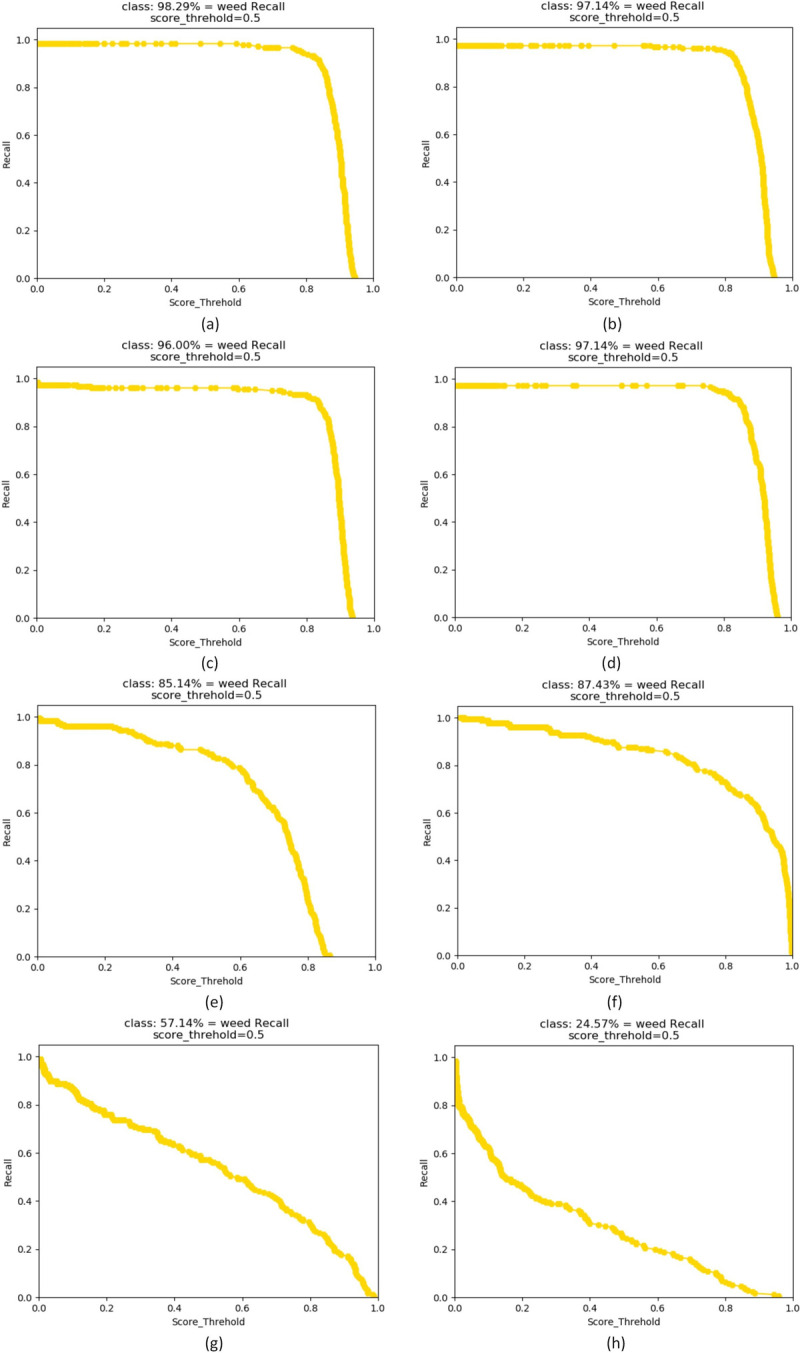
Comparison chart of recall curves of different models. (a) YOLOX-tiny; (b) YOLOX-nano; (c) YOLOX-s; (d) YOLOX-m; (e) YOLOv5-s; (f) SSD; (g) YOLOv4-tiny; (h) YOLOv3.

As shown in Fig 10, in the comparison of the recall curves of the different models, the abscissa represents the threshold value of the algorithm model for tetaining prediction boxes, and the ordinate represents the recall. This curve represents the ratio of the number of correct frames predicted by the algorithm model to the number of all real frames for a fixed threshold. When the threshold is larger, if a model can still maintain a high recall rate, this indicates that the model has a very good detection effect on the dataset. It can be seen from this figure that the YOLOX algorithm models of all sizes and the SSD model can maintain high recall rates under high threshold conditions. The next best-performing model is the YOLOv5 network, whereas the recall rates of the YOLOv4-tiny and YOLOv3 models are relatively low, indicating that they have no good generalization ability among datasets.

**Fig 11:**
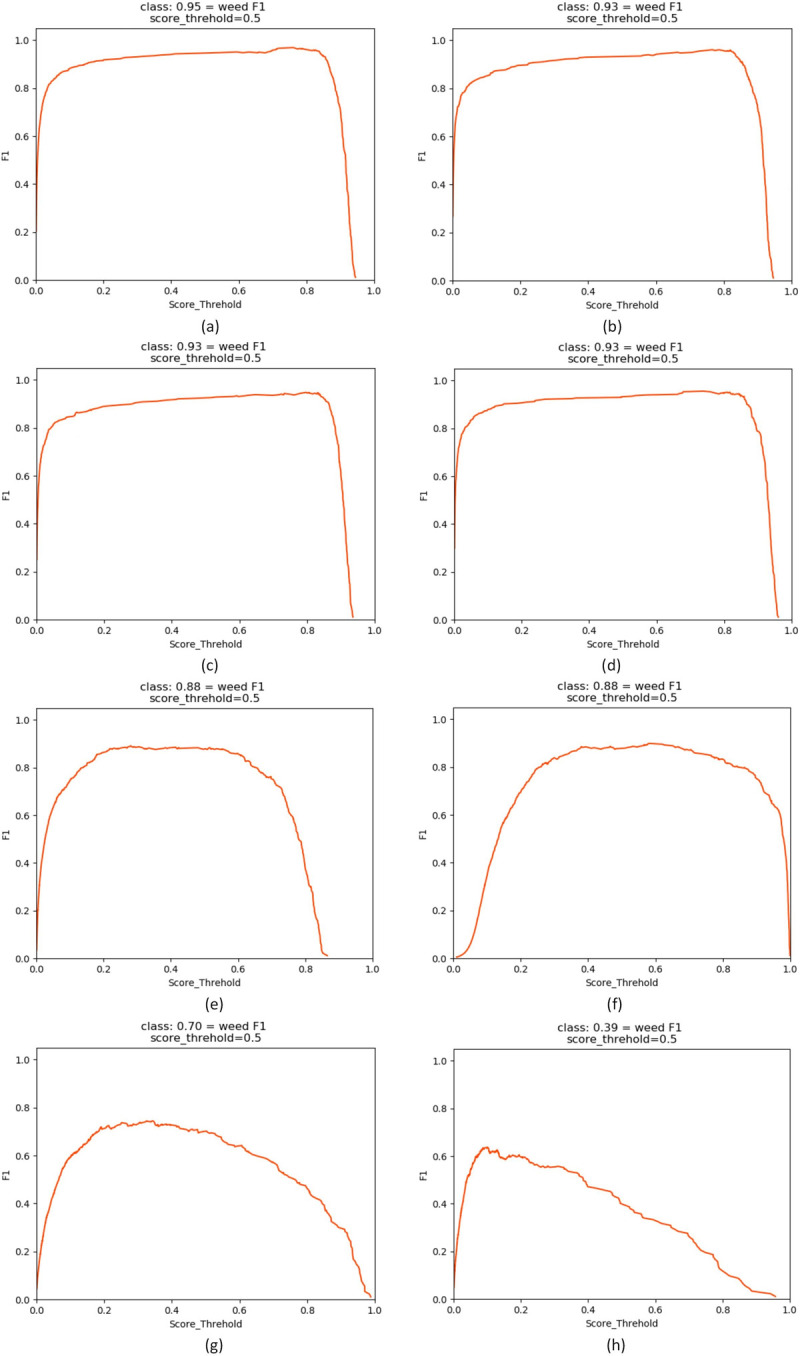
Comparison chart of F1 value curves of different models. (a) YOLOX-tiny; (b) YOLOX-nano; (c) YOLOX-s; (d) YOLOX-m; (e) YOLOv5-s; (f) SSD; (g) YOLOv4-tiny; (h) YOLOv3.

As shown in Fig 11, in the comparison of the F1 value curves of the different models, the abscissa represents the threshold value of the algorithm model for retaining prediction boxes, and the ordinate represents F1. To evaluate different algorithms, the concept of the F1 value has been proposed based on precision and recall to evaluate these factors overall as a whole. When the curve of the F1 value contains more area, the model has a better detection effect on the dataset. The YOLOX series algorithms perform the best in the F1 value curve evaluation, followed by the YOLOv5 and SSD networks, whereas the YOLOv4-tiny and YOLOv3 models are relatively poor.

### Weed detection effect based on YOLOX-tiny

**Fig 12:**
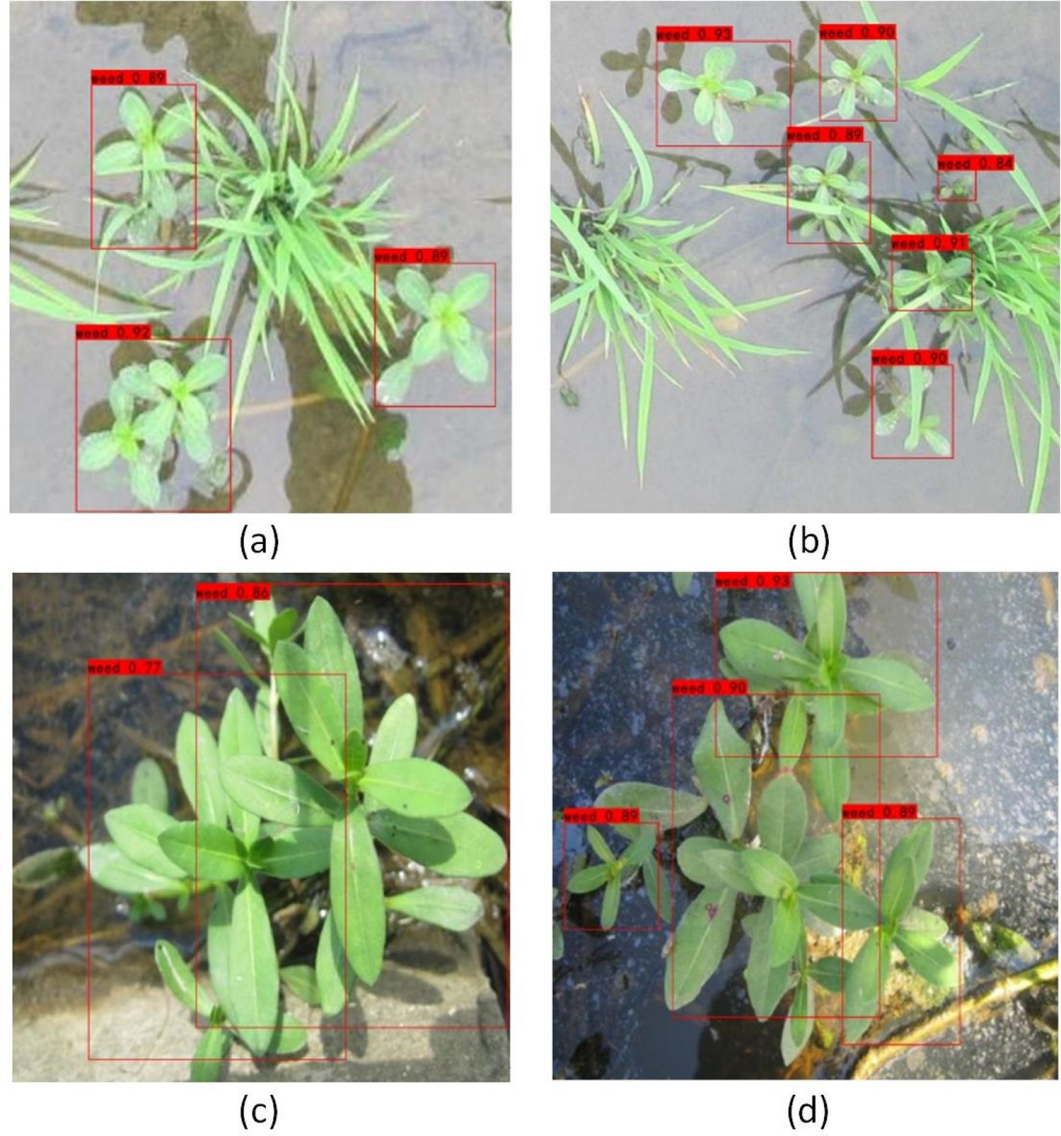
Weed target detection effects based on YOLOX-tiny. (a) partial collusion; (b) multiple weed targets and serious occlusion; (c) dense weeds; (d) dense weeds and different target sizes.

Due to different germination periods of Alternanthera philoxeroides in rice fields, different Alternanthera philoxeroides plants will be in different growth periods at the same time in the same field, and the shapes and postures of weeds in different growth periods will be different. At the same time, due to the clustered nature of these weeds, Alternanthera philoxeroides plants can seriously block each other. The detection effects of YOLOX-tiny for Alternanthera philoxeroides targets in cases of multiple targets with different occlusion degrees are shown in Fig 12. Even with multitarget occlusion and a dense weed distribution, weed targets of Alternanthera philoxeroides can be correctly detected, without missed detection or multiple detection, meaning that the YOLOX-tiny model can meet the application requirements.

## Conclusions

Under the condition of a small sample of rice field weed images, a method based on YOLOX was proposed to detect weeds in rice fields at the seedling stage, addressing the challenges of small weed target detection, such as weed occlusion, density, and inconsistent scale. A small sample dataset of lotus seed weeds in a rice field at the seedling stage was collected, and eight target detection models in total were built and trained on this dataset. Specifically, YOLOv3, YOLOv4-tiny, and YOLOv5-s models from the YOLO series; YOLOX-m, YOLOX-s, YOLOX-nano, and YOLOX-tiny models as improved variants of the YOLO algorithm and an SSD model as a representative of a classical network algorithm were tested and analyzed. A weed target detection model suitable for embedded computing platforms was identified by comparing these different single-stage models, thereby laying a foundation for the realization of unmanned targeted herbicide spraying performed by agricultural robots. As a result, it was concluded that the comprehensive performance of the YOLOX-tiny model was the best. The mAP value, F1 value and recall value of the YOLOX-tiny model were 0.980, 0.95, and 0.983, respectively. Meanwhile, the intermediate variable memory generated during the model calculation of YOLOX-tiny was sufficiently small, making this model suitable for deployment in intelligent agricultural devices. However, although the YOLOX-tiny model is the best on the dataset considered in this paper, this is not true in general. The experimental results showed that the YOLOX series models could behave well in detecting the positions of Alternanthera philoxeroides weeds in the complex environment of a rice field. The YOLOX weed target detection models can ensure the accurate and real-time detection of weed position in a rice field, provide technical support for the targeted application of herbicides in paddy fields, and serve as a reference for research on weed target detection for weed associated with other crops. The main shortcomings of our study were the small size of the weed image sample collected by a hand-held camera and the fact that the algorithms were not improved with channel attention module.
